# An anthocyanin marker for direct visualization of plant transformation and its use to study nitrogen-fixing nodule development

**DOI:** 10.1007/s10265-019-01126-6

**Published:** 2019-07-19

**Authors:** Senlei Zhang, Éva Kondorosi, Attila Kereszt

**Affiliations:** Institute of Plant Biology, Biological Research Centre, Hungarian Academy of Sciences, Temesvári körút 62, 6726 Szeged, Hungary

**Keywords:** Reporter gene, Transgenic tissue, Hairy root, Legume symbiosis, Nodule-specific cysteine-rich (NCR) peptides

## Abstract

The development and functioning of the nitrogen fixing symbiosis between legume plants and soil bacteria collectively called rhizobia requires continuous chemical dialogue between the partners using different molecules such as flavonoids, lipo-chitooligosaccharides, polysaccharides and peptides. *Agrobacterium rhizogenes* mediated hairy root transformation of legumes is widely used to study the function of plant genes involved in the process. The identification of transgenic plant tissues is based on antibiotics/herbicide selection and/or the detection of different reporter genes that usually require special equipment such as fluorescent microscopes or destructive techniques and chemicals to visualize enzymatic activity. Here, we developed and efficiently used in hairy root experiments binary vectors containing the *MtLAP1* gene driven by constitutive and tissue-specific promoters that facilitate the production of purple colored anthocyanins in transgenic tissues and thus allowing the identification of transformed roots by naked eye. Anthocyanin producing roots were able to establish effective symbiosis with rhizobia. Moreover, it was shown that species-specific allelic variations and a mutation preventing posttranslational acetyl modification of an essential nodule-specific cysteine-rich peptide, NCR169, do not affect the symbiotic interaction of *Medicago truncatula* cv. Jemalong with *Sinorhizobium medicae* strain WSM419. Based on the experiments, it could be concluded that it is preferable to use the vectors with tissue-specific promoters that restrict anthocyanin production to the root vasculature for studying biotic interactions of the roots such as symbiotic nitrogen fixation or mycorrhizal symbiosis.

**Electronic supplementary material:**

The online version of this article (10.1007/s10265-019-01126-6) contains supplementary material, which is available to authorized users.

## Introduction

Genetic transformation of plants plays an essential role both in basic research to reveal the function of genes/proteins and in biotechnology to improve crop yield by introducing, knocking out or modifying certain genes and/or pathways. To obtain transgenic plants, however, is a laborious and time-consuming process and is not successful in all plant species/genotypes (Delporte et al. 2012). It requires the introduction of foreign genes either by *Agrobacterium* mediated or direct DNA transfer methods (Fraley et al. 1986; Miao and Jiang 2007; Taylor and Fauquet 2002), the selection and culturing of the transgenic tissues and finally, the regeneration of the whole plants carrying the transgene(s). Instead of this tedious and long procedure, composite plants comprising transgenic “hairy roots” on untransformed shoots (Chandra 2012) can be obtained in significantly shorter time via *Agrobacterium rhizogenes* mediated transformation. The use of transformed hairy roots contributed to many significant achievements in molecular biology, and particularly in symbiotic nitrogen fixation.

Legumes are able to establish symbiotic association with soil bacteria collectively called rhizobia to secure their nitrogen need via the reduction of atmospheric nitrogen into ammonium. In this association, the plant partner provides a proper niche as well as energy and carbon source for the bacterial partner, which, in turn, supplies the plant with the reduced nitrogen. Symbiotic nitrogen fixation takes place in a newly formed symbiotic organ, the root nodule. Nitrogen fixing nodule development is based on continuous exchange of signal molecules that are required for the recognition of the partners, initiation of nodule organogenesis, infection of plant cells with the Rhizobium bacterial partner and differentiation of nodule cells and conversion of bacteria to nitrogen fixing bacteroids (Gibson et al. 2008; Oldroyd et al. 2011). In certain legumes, such as *Medicago*, *Pisum*, *Trifolium* species of the IRLC (Inverted Repeat Lacking Clade), bacteroid differentiation is irreversible and is associated with genome amplification, increase of cell size and membrane permeability as well as loss of cell division capacity (Kereszt et al. 2011; Mergaert et al. 2006). This terminal differentiation is controlled by the plant via nodule-specific cysteine-rich (NCR) peptides that are almost exclusively produced in the infected nodule cells and are targeted to the bacterial cells (Durgo et al. 2015; Guefrachi et al. 2014; Montiel et al. 2016, 2017; Van de Velde et al. 2010). Legumes of the IRLC produce variable number of NCRs (from seven in *Glycyrrhiza* to over 700 in *Medicago*) that act in successive waves and alter progressively the physiology and morphology of the intracellular bacteria (Farkas et al. 2014; Horvath et al. 2015; Kim et al. 2015; Montiel et al. 2017; Van de Velde et al. 2010). Despite the huge number of peptides and the expected functional redundancy, certain peptides such as NCR169 and NCR211—missing from the *dnf7* (Horvath et al. 2015) and *dnf4* (Kim et al. 2015) mutants, respectively—are essential for bacteroid and nodule development in *Medicago*.

Hairy root transformation of legumes (Boisson-Dernier et al. 2001; Clemow et al. 2011; Estrada-Navarrete et al. 2007; Kereszt et al. 2007; Stiller et al. 1997) has been widely used in symbiosis research, for example, to complement symbiotic mutants (Endre et al. 2002; Indrasumunar et al. 2010, 2011; Madsen et al. 2003), to reveal the spatial and temporal aspects of gene expression and to identify promoter elements (Bersoult et al. 2005; Gavrilovic et al. 2016; Liu et al. 2019), to determine the cellular localization of proteins (Gavrin et al. 2014, 2017; Limpens et al. 2009), to overexpress (Indrasumunar et al. 2011; Reid et al. 2011), to silence (Limpens et al. 2004, 2005; Sinharoy et al. 2015; Sogawa et al. 2018) or to knock-out genes (Michno et al. 2015; Wang et al. 2017, 2018). Despite its many advantages, the efficiency of hairy root transformation is not 100% even with antibiotic or herbicide selection, i.e. not all the roots formed on transformed plants are transgenic. The identification of transgenic tissues via the detection of the proteins produced by the currently used reporter genes coding for β-glucuronidase/GUS in vectors such as pBI121 or the pCAMBIA series (Jefferson et al. 1986, 1987; https://cambia.org/welcome-to-cambialabs/cambialabs-projects/cambialabs-materials-and-methods-developed-in-cambialabs/) and fluorescent proteins (GFP, YFP, DsRed, etc.) in plasmids like the pUB series or the pHairyRed (Lin et al. 2011; Maekawa et al. 2008) requires destructive techniques and fluorescent microscopes, respectively, making the screening impossible or uncomfortable if further tissue growth is needed subsequently. Therefore, an easy, non-destructive identification of transgenic roots is highly needed.

Here, we describe a new set of binary transformation vectors (pPurpleRoot) developed from the widely used pCAMBIA plasmids that allow the identification of transgenic tissues by naked eye without the use of staining techniques or fluorescent microscopy. They contain the *MtLAP1* gene coding for an R2R3 type MYB transcription factor (Peel et al. 2009) that induces anthocyanin production providing purple coloration for the T-DNA harbouring tissues in the transgenic plants. The MtLAP1 protein was shown to direct the production and accumulation of the anthocyanins in *Medicago truncatula*, *M. sativa* and *Trifolium repens* as well as with a lower intensity and homogeneity in tobacco after transient (Picard et al. 2013) or ectopic (Peel et al. 2009) expression.

Moreover, the vectors were successfully tested for the genetic complementation of the nodule developmental defect of the *M. truncatula dnf7* mutant via *A. rhizogenes* mediated transformation. The complementation experiments showed that species-specific allelic variations (*Melilotus albus* vs. *Medicago truncatula*) and a mutation (K40R) preventing posttranslational acetyl modification of an essential nodule-specific cysteine-rich peptide, NCR169, do not affect the symbiotic interaction of *Medicago truncatula* cv. Jemalong with *Sinorhizobium medicae* strain WSM419.

## Materials and methods

### Biological materials, growth conditions and phenotypic assays

*Escherichia coli* strain MDS™42 ΔrecA Blue (Scarab Genomics, USA) was used for cloning purposes and grown at 37 °C in LB medium (10 g l tryptone^−1^; 5 g l yeast extract^−1^; 5 g l NaCl^−1^). *Agrobacterium rhizogenes* strain ARqua (Quandt et al. 1993) harboring the different binary vectors and grown in LB medium at 30 °C was used to induce hairy roots. Wild-type and *dnf7*-*2* (Horvath et al. 2015) mutant *Medicago truncatula* cv. Jemalong (Register of Australian Herbage Plant Cultivars: Reg. No. B-9a-2) plants were inoculated with *Sinorhizobium medicae* strain WSM419 (Reeve et al. 2010) that was grown in TA medium (10 g l tryptone^−1^; 1 g l yeast extract^−1^; 5 g l NaCl^−1^; 1 mM MgSO_4_; 1 mM CaCl_2_) at 30 °C for 2 days. *M. truncatula* plants were germinated on water-agar plates at 24 °C in dark. Plants were grown in vermiculite and assayed for nitrogen fixation ability in a glasshouse at 22 °C with 16/8 h light/dark cycles. Nitrogen fixation efficiency of all plants was assessed by both the phenotype (green healthy Fix^+^ plants versus nitrogen starved yellow Fix^−^ plants) and the dry weight of the plants 6 weeks after inoculation. Dry weight measurement was performed after drying the samples at 80 °C for 48 h. β-Glucuronidase activity was detected according to Jefferson et al. (1987).

### Vector and gene constructions

The *MtLAP1* gene was amplified with Phusion High-Fidelity DNA Polymerase (ThermoFisher Scientific) using *M. truncatula* cv. Jemalong genomic DNA as template and the MtLAP1_pCncoF-MtLap1_pCeheR primer pair (Table 1) and the PCR fragment was cloned into the *Nco*I–*Ehe*I digested pCAMBIA2201 vector with the help of the In-Fusion Ligation Kit (TaKaRa) to replace the *gusA* gene coding for β-glucuronidase. To provide other regulatory sequences, first, the Cauliflower Mosaic Virus 35S promoter was removed and the promoter sequences of the *At2g37950* (*pAtE47*) and the *At5g24800* (*pAtS5*) genes showing tissue-specific expression in both *Arabidopsis thaliana* (Lee et al. 2006) and *Lotus japonicus* (Gavrilovic et al. 2016) were amplified using *Arabidopsis thaliana* ecotype Columbia (Rédei 1992) genomic DNA as template and primer pairs 2g37950prF-2g37950prR and 5g24800prF-5g24800prR were cloned at the NcoI site with the help of the In-Fusion Ligation Kit (TaKaRa). The vectors harboring the CaMV 35S, pAtE47 and pAtS5 promoters in front of the MtLAP1 gene were named pPurpleRootC, pPurpleRootE and pPurpleRootP, respectively.

**Table 1:** Oligonucleotides used for the construction of vectors and *NCR169* alleles

Primer	Sequence
MtLAP1_pCncoF	5′-GGACTCTTGACCATGGAGAATACCGGAGGTGTGAG-3′
MtLap1_pCeheR	5′-ACCTGTAATTCACACGTGTCAAGGTAGATCCCAAAG-3′
2g37950prF	5′-AGCAGCTTGACCATGGGCCACCAGCCAAATGTTTCTG-3′
2g37950prR	5′-TCCGGTATTCTCCATGATTTTTGCCTAATGAATGTTTCTTTTTG-3′
5g24800prF	5′-AGCAGCTTGACCATGGCTACGTATAGTGGATATACGTCGTTCC-3′
5g24800prR	5′-TCCGGTATTCTCCATGTTCTTTGAATGTGAACACACAAGAAAGA-3′
MaNCR169salF	5′-AAGTCGACAAGATGGTTTAGTACATC-3′
MaNCR169HindR	5′-CCAAGCTTATACCAGAGAACGCAAATATTTTC-3′
NCR169K40Rrev	5′-AGATCTGTAACAATCATCAACAATACC-3′
NCR169K40Rfw	5′-GATTGTTACAGATCTAAGAAACCTCTTTTTAAAATTTGG-3′
NCR169sacF	5′-GAAAGGTTGTTAAACAATAATGAG-3′
NCR169hindR2	5′-AAAAGCTTATACCAAAGAACACAAACA-3′
lacZncoR	5′-ATCCATGGTCAAGCTGCTCTAGCATTC-3′

The primer pair MaNCR169salF-MaNCR169HindR was used to amplify the *NCR169* gene of *M. albus*, then the *Sal*I–*Hin*dIII fragment replaced the *Medicago* sequence in the *M. truncatula NCR169*-*Strep* construct (Horvath et al. 2015). To introduce the K40R mutation into the *MtNCR169* gene, overlapping fragments were amplified with the help of the NCR169sacF-NCR169K40Rrev and the NCR169K40Rfw-NCR169hindR2 primer pairs that were joined by overlapping PCR. The mutant fragment replaced the wild-type fragment in the *NCR169*-*Strep* construct.

### *Agrobacterium rhizogenes* mediated transformation

The “agar only” method followed the protocol described by Boisson-Dernier et al. (2001), however, no kanamycin selection was applied. In short, the root tip of young radicles of 8–10 mm length was excised and the cut surface was immersed in agrobacteria grown on plates. *Agrobacterium* infected plants were kept on Buffered Nod Medium (Ehrhardt et al. 1992) agar plates in a humid chamber at 22 °C with 16/8 h light/dark cycles until roots of approximately 2–3 cm long were formed. Transformed wild-type plants were inoculated with bacteria on Buffered Nod Medium agar plates, while transformed *dnf7* mutants were transferred into vermiculite and were inoculated with rhizobia 1 week after transfer. Reporter gene activity was observed before and 6 weeks after inoculation.

For the “soil plug” method, the sectioned radicles of *dnf7* plants were coated with agrobacteria the same way as during the agar only protocol and kept on agar plates for 3 h at 22 °C. Next, the treated seedlings were transferred into soil of small particle size placed into 1 cm long tubes (diameter 8 mm) that were kept in a humid chamber at 22 °C with 16 h photoperiod for 2 weeks. The *dnf7* seedlings that formed roots in the soil plugs were transferred into vermiculite for further analysis. Reporter gene activity was observed 6 weeks after inoculation.

## Results and discussion

### Construction of the pPurpleRoot vectors

To facilitate the selection of transgenic roots without the use of fluorescent microscopy or destructive staining techniques to detect fluorescence (e.g. GFP,) or enzyme activity (e.g. β-glucuronidase), respectively, from reporter proteins, we constructed binary vectors by replacing the CaMV 35S promoter-GUS reporter gene of pCAMBIA3301 by the exons and introns of the *MtLAP1* gene (Peel et al. 2009) driven by different promoters. The MtLAP1 transcription factor belongs to the largest and plant/yeast specific R2R3 subfamily of the MYB transcription factors that play important roles in the transcriptional regulation of anthocyanin biosynthesis (Naing and Kim 2018). Transient (Picard et al. 2013) or ectopic (Peel et al. 2009) expression of *MtLAP1* results in anthocyanin production and accumulation in *Medicago truncatula*, *M. sativa* and *Trifolium repens* as well as with a lower intensity and homogeneity in tobacco. For constitutive expression of this transcription factor, the Cauliflower Mosaic Virus 35S (*CaMV35S*) promoter driven *gusA* gene coding for the β-glucuronidase enzyme was replaced with the coding and intron sequences of the *MtLAP1* gene in the pCAMBIA2201 binary vector and the resulting plasmid was named as pPurpleRootC (constitutive). As anthocyanins might have antimicrobial activity and its constitutive production might affect the rhizobium–plant interaction, the constitutive *CaMV35S* promoter of pPurpleRootC was replaced by two tissue-specific promoters from *Arabidopsis* (Lee et al. 2006), namely by *pAtS5* (*At5g24800*) and *pAtE47* (*At2g37950*) that in *Lotus japonicus* provided pericycle/phloem and endodermis/pericycle enriched promoter activities, respectively (Gavrilovic et al. 2016). These plasmids were named pPurpleRootP (pericycle/phloem) and pPurpleRootE (endodermis/pericycle). The general outline of the vectors is shown in Fig. S1.

### Anthocyanin reporter allows the easy visual selection of transgenic roots and the formation of effective root nodules

**Fig. 1:**
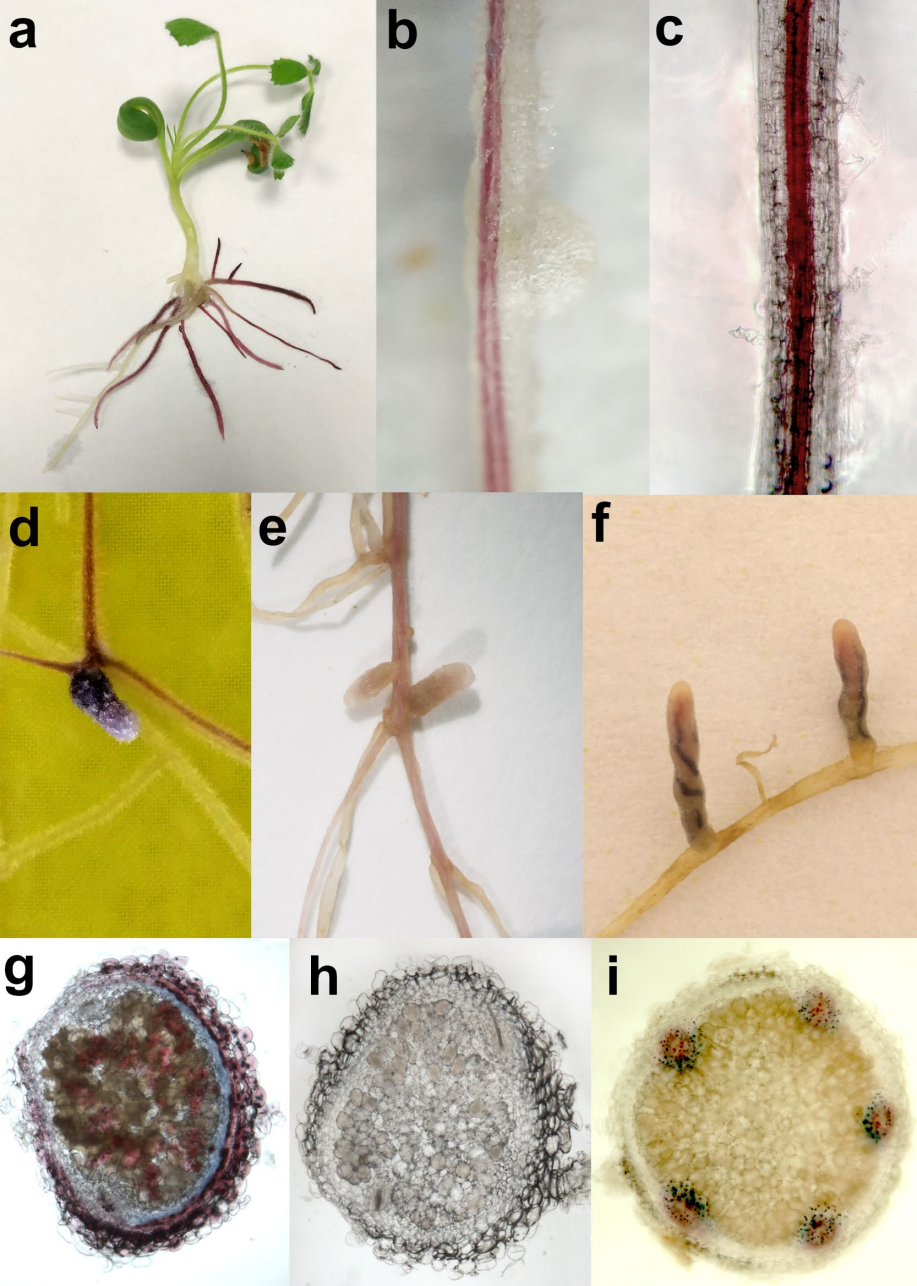
Anthocyanin production in the transgenic tissues. **a** Transgenic hairy roots formed on *M. truncatula* shoots display purple coloration. **b** Endodermis/pericycle and **c** Pericycle/phloem specific production of anthocyanins after transformation with pPurpleRootE and pPurpleRootP, respectively. **d**, **g** Strong coloration of the vascular and nodule tissues of roots formed after transformation with pPurpleRootC. **e**, **h** Nodules formed on pPurpleRootE transformed roots do not produce anthocyanins in the nodule (vasculature). Note that no coloration can be observed in young developing nodules in **b** either. **f**, **i** Anthocyanin accumulation in the nodule vasculature on hairy roots transformed with pPurpleRootP. Note that weak or no coloration can be observed in the root vasculature of older plants (at least 6 weeks after inoculation) shown in **e** and **f**

To test the usability of the anthocyanin reporter system, the pPurpleRoot vectors were transformed into *A. rhizogenes* strain ARqua1, and then the bacteria were used to induce the formation of transgenic hairy roots on *M. truncatula* cv. Jemalong A17 plants. The purple color of roots due to anthocyanin production made it easy to distinguish the transgenic roots from the non-transformed ones (Fig. 1). Roots transformed with the pPurpleRootC construct showed strong coloration in the whole root that was even more enhanced in the vascular tissues (Fig. 1a, d). Coloration of the transgenic roots emerging after transformation with pPurpleRootE (Fig. 1b) and pPurpleRootP (Fig. 1c) vectors was in agreement with the expected expression pattern in the endodermis/pericycle and pericycle/phloem tissues, respectively.

To investigate whether the production of anthocyanins affects the development of symbiotic nodules, the transgenic roots were inoculated on agar plates with *Sinorhizobium medicae* strain WSM419. Although these environmental conditions allowed the formation of only low numbers of nodules that have deep purple coloration (most probably, as the mixture of leghemoglobin and anthocyanin colors) or purple vascular bundles on the roots transformed with pPurpleRootC (Fig. 1d, g) and pPurpleRootP (Fig. 1f, i) vectors, respectively, while no coloration of nodules could be observed on pPurpleRootE transformed roots (Fig. 1b, e, h).

**Fig. 2:**
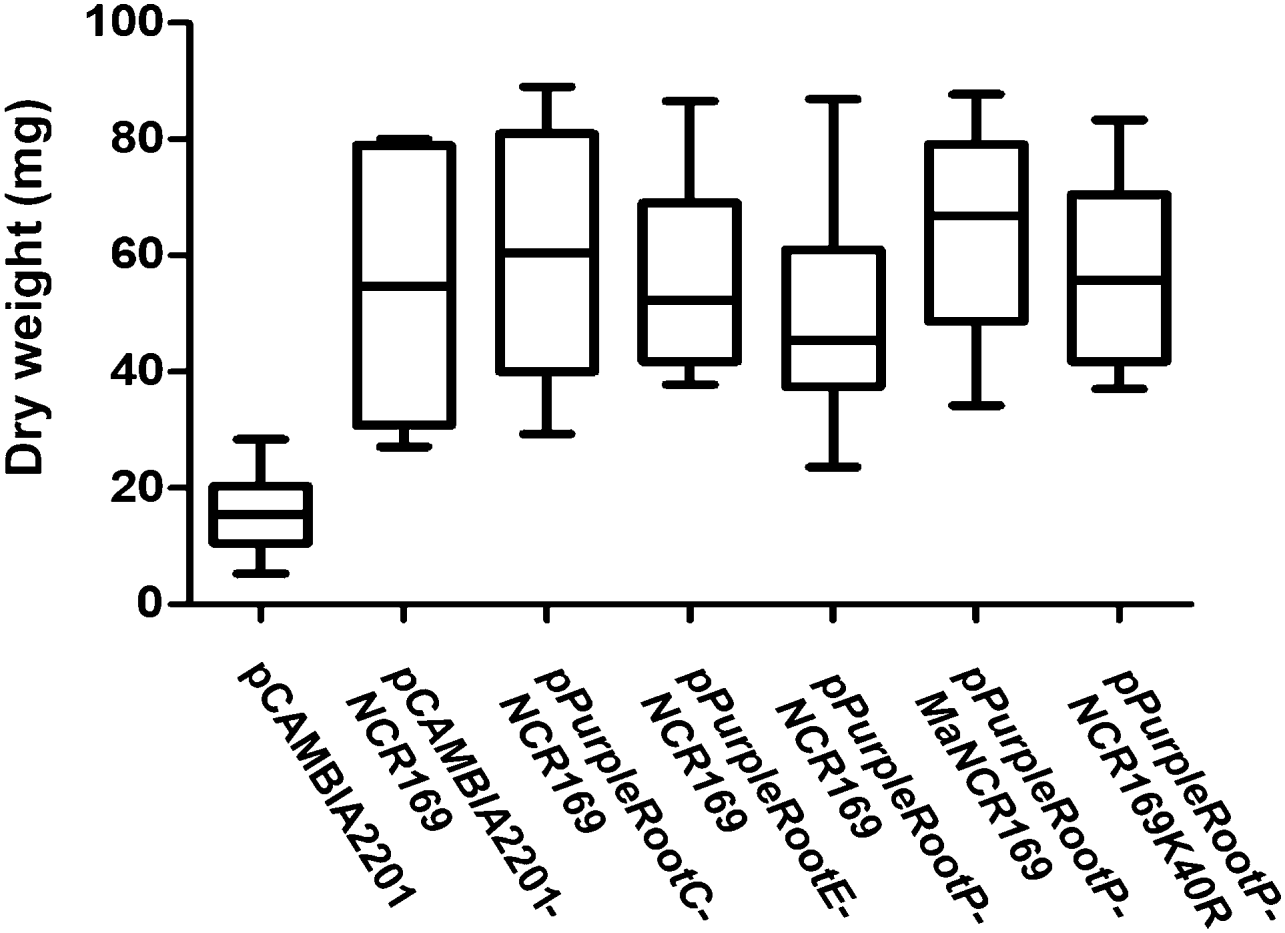
Dry weigth of *dnf7*-*2* mutant plants carrying transgenic roots after transformation with the indicated vector constructs

The usability of this new reporter/vector system in symbiosis was tested by complementation of the *M. truncatula dnf7*-*2* mutant (Table 2; Fig. 2) carrying a deletion in the *ncr169* gene and resulting in Fix^−^ phenotype (Horvath et al. 2015). To compare the efficiency of the vectors, the *ncr169* gene was cloned both into the initial pCAMBIA2201 and into the pPurpleRoot vectors. The constructs were introduced into the mutant with the two different transformation protocols described in the “Materials and methods” section in details, and then the transformed plants were transferred into vermiculite where nodulation assays were performed. The plants were inoculated with *S*. *medicae* strain WSM419 and their root/nodule and symbiotic phenotypes were evaluated 6 weeks post-inoculation. In the “agar only” experiment, reporter gene activity driven by the constitutive CaMV35S promoter was observed (Table 2) in at least on one root of almost all plant (pCAMBIA2201: 90–95%; pPurpleRootC: 100%). In the case of pPurpleRootE and pPurpleRootP transformed roots, anthocyanin accumulation indicated lower numbers of transformed plants corresponding to ~ 58% and ~ 44% transformation frequency, respectively. However, there were pPurpleRootE and pPurpleRootP transformed plants in which anthocyanin could not be seen in the roots, though they were complemented as manifested in green foliage and high biomass (Fig. 2). One possible explanation for this observation is that reporter gene activity might have been silenced via post-transcriptional gene silencing (Depicker and Van Montagu 1997) resulting in no or too low anthocyanin accumulation to detect in these tissues. The complementation efficiency of the pPurpleRootE and pPurpleRootP constructs was comparable to that of the pCAMBIA2201 harbouring the *ncr169* gene. In contrast, despite the high transformation rate, the complementation efficiency of the pPurpleRootC derived construct was very low (~ 20%). Although antimicrobial activity and the mode of actions of anthocyanins has been studied and shown mostly in relation to human health (reviewed in Smeriglio et al. 2016), these results indicate that too high level of anthocyanins may negatively affect the interaction of the plant roots with rhizobia. Interestingly, in the “soil plug” experiment, the ratio of plants with roots expressing the transgene from the 35S promoter was lower than when the “agar only” method was used (Table 2), but all the plants scored to be transgenic were complemented. The explanation for this observation requires further experiments.

**Table 2:** Transformation and complementation efficiency by using the pPurleRoot vectors

Construct	Number of plants with visible reporter gene activity (total number of plants)	Number of plants with effective nodules showing reporter gene activity	Number of plants with effective nodules lacking reporter gene activity
pCAMBIA2201^a^	19 (20)	0	0
pCAMBIA2201::NCR169^a^	18 (20)	12	0
pPurpleRootC::NCR169^a^	20 (20)	5	0
pPurpleRootE::NCR169^a^	11 (19)	11	5
pPurpleRootP::NCR169^a^	7 (16)	7	3
pCAMBIA2201^b^	74 (80)	0	0
pCAMBIA2201::NCR169^b^	76 (78)	38	0
pPurpleRootC::NCR169^b^	20 (80)	20	0
pPurpleRootE::NCR169^b^	36 (79)	36	ND
pPurpleRootP::NCR169^b^	29 (77)	29	ND

^a^Co-cultivation and hairy root development on agar plates

^b^Hairy root development in soil plugs

### Allelic and lysine-acetylation variations do not affect the symbiotic activity of the NCR169 peptide

Allelic variations and post-translational modifications (PTMs) in proteins may affect their biological activity. Indeed, allelic variations of certain NCR peptides have been shown to affect the interaction of the host with certain strains of their bacterial partners (Wang et al. 2017, 2018; Yang et al. 2017). Similarly, it was shown that the NCR169 peptide essential for bacteroid development and symbiotic nitrogen fixation (Horvath et al. 2015) carries an acetyl modification at lysine 40 in *M. truncatula* nodules (Marx et al. 2016). This reversible type of PTM changes the charge of proteins and their interactions with other macromolecules as best known in the case of histones (Drazic et al. 2016). The mature NCR169 peptides of *M. truncatula* and *Melilotus albus* share 68% amino acid identity (Horvath et al. 2015). One of the differing residues is an asparagine in the *Melilotus* peptide in place of the lysine (K40) acetylated in *Medicago*, however, this amino acid has similar characteristics (polar, uncharged side chain) as the acetylated lysine, thus, most probably, it does not affect activity. To investigate whether the other amino acid differences in the *Melilotus ncr169* allele affect the complementation of the *M. truncatula* mutant and whether the lysine acetylation of NCR169 is required for its biological activity, the *ncr169* gene of *M. albus* (*MaNCR169*) and a mutant *Medicago* gene coding for a peptide in which the acetylated residue is replaced by the positively charged arginine (NCR169K40R) were cloned into pPurpleRootP and were tested to complement the *dnf7*-*2* mutant. Both constructs were able to restore the symbiotic nitrogen fixation capability of the mutant (Fig. 2) indicating that NCR169 of *M. albus* have the same biological activity as that of *M. truncatula* cv. Jemalong during the interaction with *S. medicae* strain WSM419 and that reversible lysine acetylation, i.e. the charge of the peptide at that position does not affect its function and activity.

## Conclusions

The facts that (1) anthocyanin accumulation could be easily observed in young hairy roots while transgene activity could not be detected with 100% probability in old tissues; and (2) roots overexpressing the *MtLAP1* gene from the pPurpleRootC vector are less efficient in developing symbiotic association have implications for the applicability of these vectors: (1) it is preferable to use the pPurpleRootE and pPurpleRootP vectors because they direct an anthocyanin accumulation that is restricted to the vasculature as well as the encircling pericycle/endodermis and is not too high. (2) Although the vectors were tested for rhizobial symbiosis, they must be also useful for research on arbuscular mycorrhizal interactions that take place on the epidermis and in the cortex of the root (Parniske 2008) where no visible anthocyanin accumulation can be observed after transformation with pPurpleRootE and pPurpleRootP. (3) The most efficient use of this reporter system is to select transgenic plants and roots with the “agar only” technique before further treatments such as bacterial or fungal inoculations are applied or hairy root cultures are established, however, when simple complementation test should be performed, the “soil plug” method is simpler and less tedious.

## Electronic supplementary material

Below is the link to the electronic supplementary material.

Supplementary material 1 (PDF 160 kb)
