# Supplementary material for: An anthocyanin marker for direct visualization of plant transformation and its use to study nitrogen-fixing nodule development
Source: J Plant Res. 2019 Jul 19;132(5):695–703. doi: 10.1007/s10265-019-01126-6 (PMC6713694; doi:10.1007/s10265-019-01126-6)
Supplement: Supplementary file 1 — Supplementary material 1 (PDF 160 kb) [file 10265_2019_1126_MOESM1_ESM.pdf]

Electronic supplementary materials

Title:

An anthocyanin marker for direct visualization of plant transformation and its use to study nitrogen-fixing nodule development

Authors:

Senlei Zhang, Éva Kondorosi, Attila Kereszt

Journal:

Journal of Plant Research

Corresponding author:

Attila Kereszt

Institute of Plant Biology, Biological Research Centre HAS

Temesvári körút 62.

6726 Szeged

Hungary

Phone number: +36-62-599672

+36-30-5062049

Fax number: +36-62-433434

E-mail: kereszta@gmail.com

Content:

Figs. S1

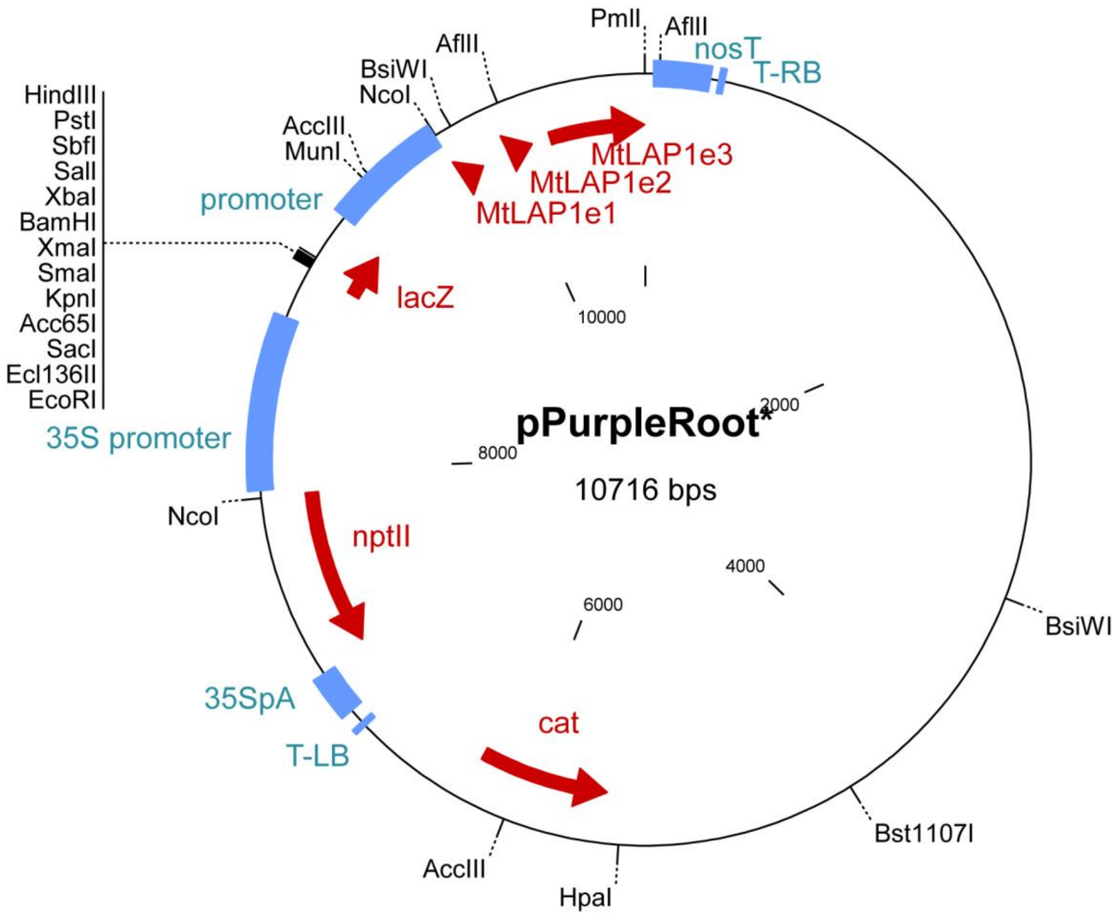

Fig. S1. General structure of pPurpleRoot vectors. Regulatory elements and coding sequences are shown in blue and red, respectively. The *cat* gene provides chloramphenicol resistance in bacteria. T-LB and T-RB denote the T-DNA left and right borders, respectively. Selection of transgenic plant tissues by kanamycin/neomycin is provided by the *nptII* gene driven by the 35S promoter and terminated by the 35S polyadenylation (35SpA) signal. The three exons of the *MtLAP1* gene (MtLAP1e1, e2, e3) is expressed from one of the three promoters, p35S, pAtE47 and pAtS5, respectively, in the three pPurpleRoot vectors. Genes of interest can be cloned at the unique cloning sites in the gene (lacZ) coding for the alpha fragment of the β-galactosidase.
